# Supplementary material for: Efficient Simultaneous Introduction of Premature Stop Codons in Three Tumor Suppressor Genes in PFFs via a Cytosine Base Editor
Source: Genes (Basel). 2022 May 7;13(5):835. doi: 10.3390/genes13050835 (PMC9140995; doi:10.3390/genes13050835)
Supplement: Supplementary file 1 [file genes-13-00835-s001.zip › Supplementary Figures S1-S5.pdf]

Supplementary information

Efficient simultaneous introduction of premature stop codons in three tumor suppressor genes in PFFs via a Cytosine Base Editor

Haoyun Jiang[1], Qiqi Jing[1], Qiang Yang[1], Chuanming Qiao[1], Yaya Liao[1], Weiwei Liu[1*] and Yuyun Xing[1*]
1State Key Laboratory of Pig Genetic Improvement and Production Technology,Jiangxi Agricultural University, Nanchang 330045, China

*Corresponding author: Tel: +86 0791-83813080, Fax: +86 0791-83813080, E-mail: xing-yuyun9@hotmail.com

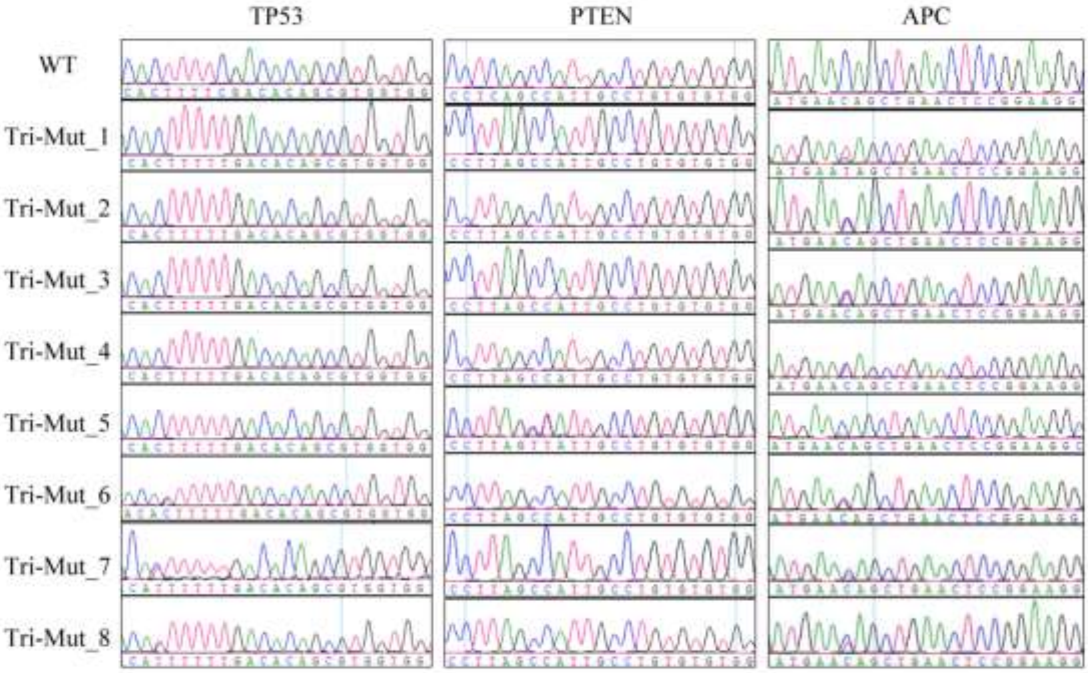

Supplementary Figure S1: Sequencing chromatograms of genotypes for 8 Tri-Mut colonies and a WT colony.

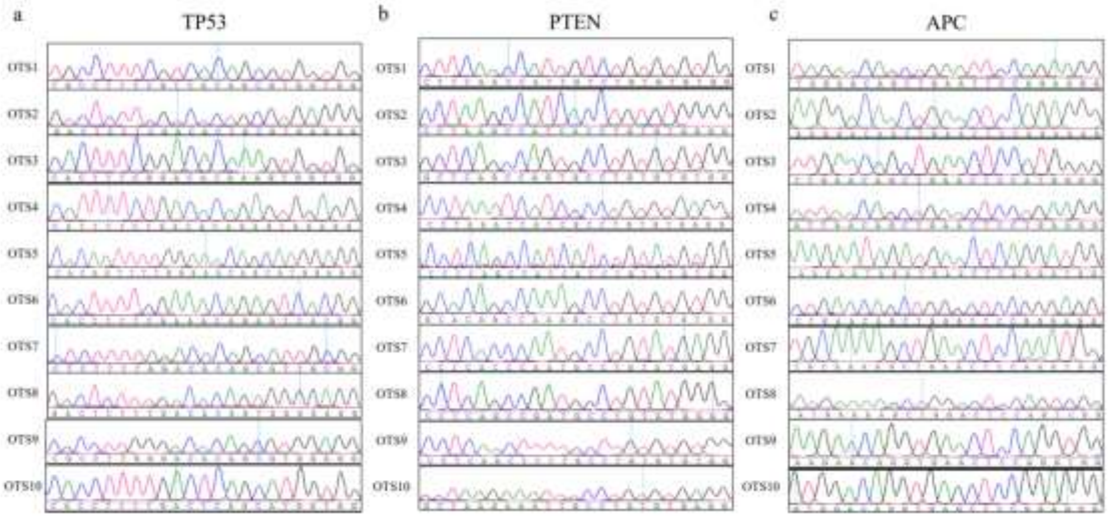

Supplementary Figure S2: Sequencing chromatograms for the predicted OTS in Tri-Mut cells. Sequencing of 10 potential OTS for TP53 (a), PTEN (b) and APC (c) sgRNAs using PCR products of 8 Tri-Mut single cell clones.

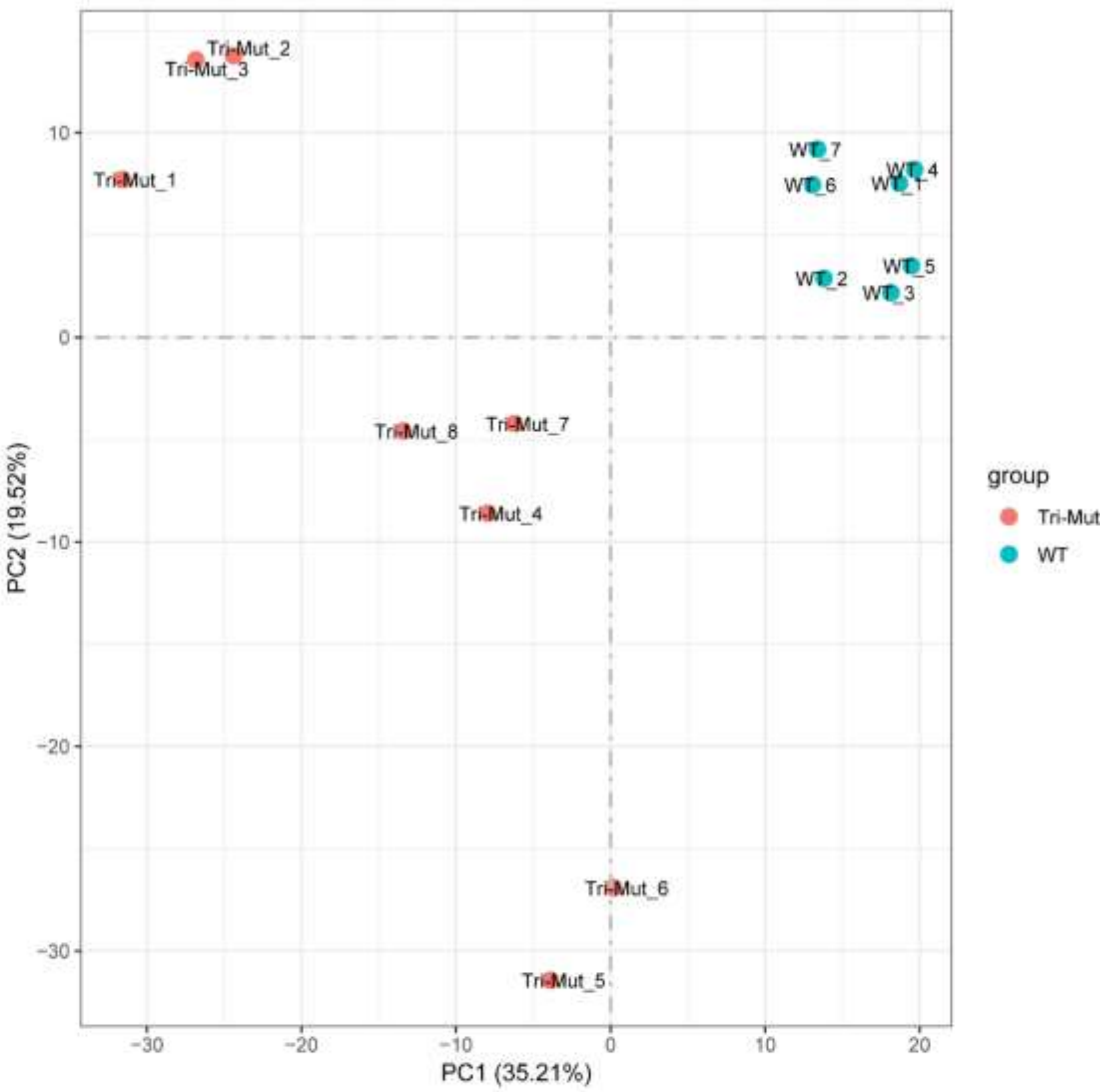

Supplementary Figure S3: Evaluation of differences and intra-group repetition. Scatter plot of the first two principal component vectors of the gene expression profiles of samples from 8 Tri-Mut single-cell clones and 7 WT single-cell clones, which are highlighted using two different colors.

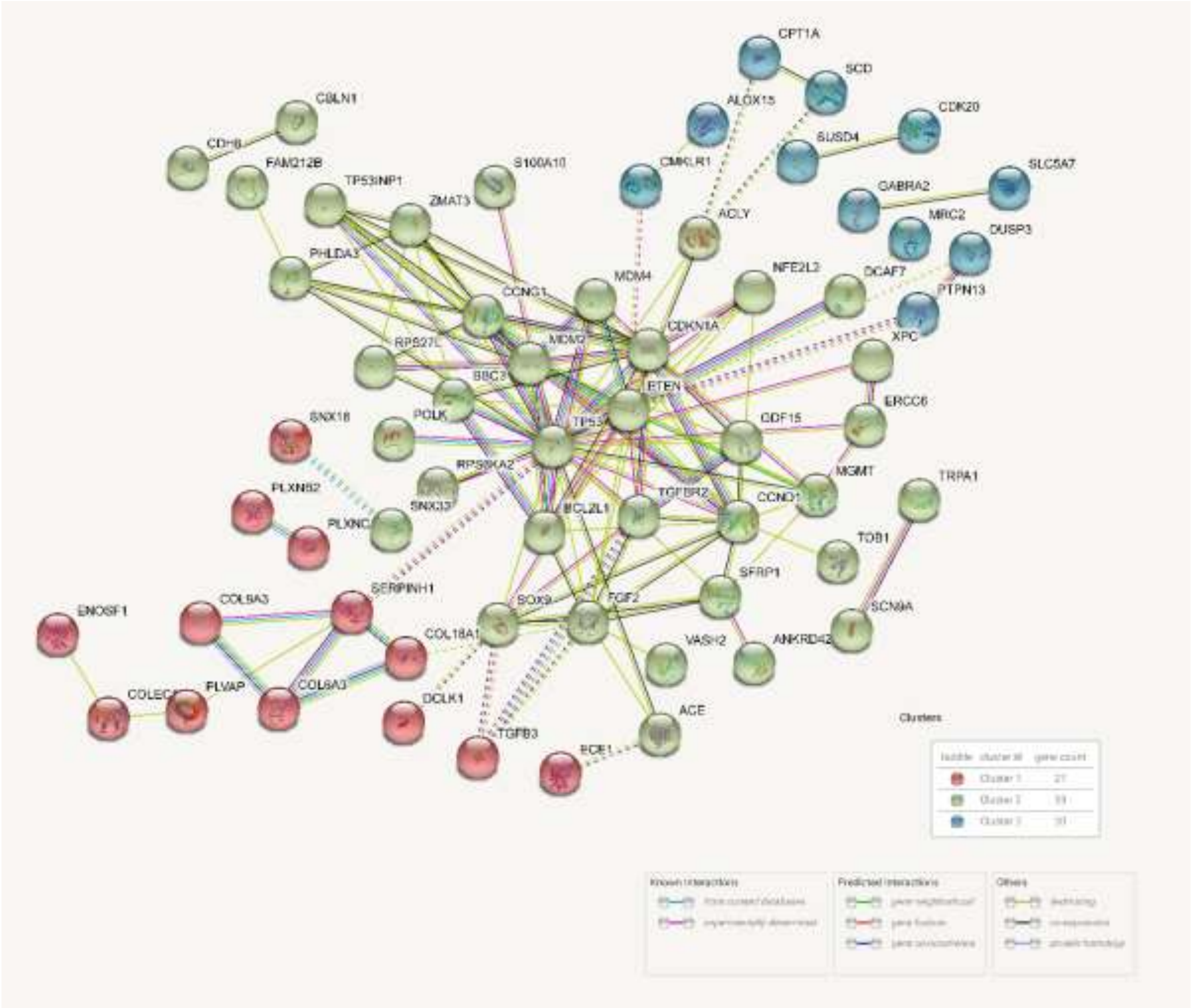

Supplementary Figure S4: Protein–protein interaction network of top 100 DEGs were analyzed by STRING analysis. Nodes represents proteins coded by DEGs and lines between nodes refers to edges. Various types of interactions are denoted by lines of different colors and defined in the legends in the figure; stand-alone nodes (nodes lacking edges) are removed.

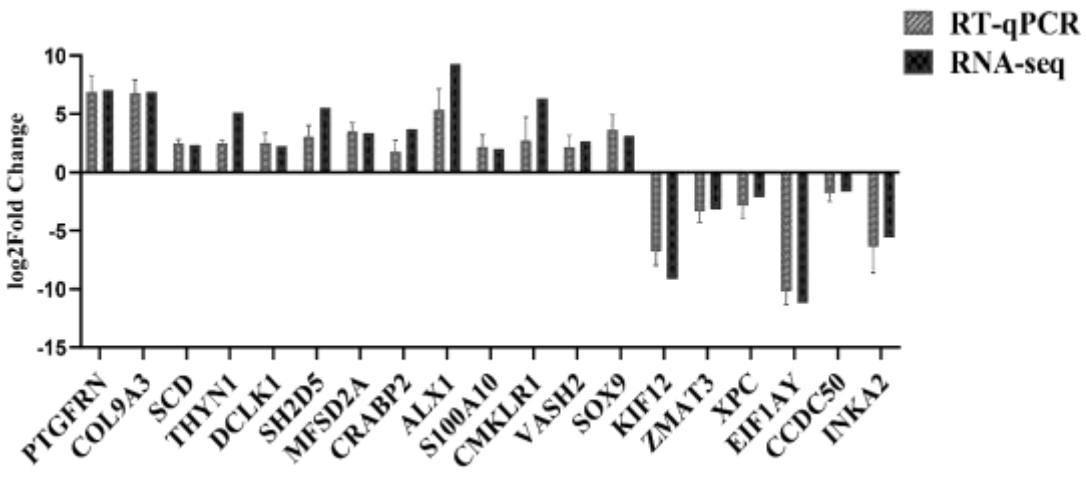

Supplementary Figure S5: Validation of expression profiling data by RT-PCR. The log2Fold Change determined from the relative Ct values of 13 up-regulated DEGs and 6 down-regulated DEGs from the Tri-Mut cell were compared to those detected by RNA-seq method. Replicates (n=8) of each sample were run and the Ct values averaged. All Ct values were normalized to β-actin. Data presented as mean ± SEM.
